# Supplementary material for: Consideration of stiffness of wall layers is decisive for patient-specific analysis of carotid artery with atheroma
Source: PLoS One. 2020 Sep 29;15(9):e0239447. doi: 10.1371/journal.pone.0239447 (PMC7523976; doi:10.1371/journal.pone.0239447)
Supplement: S1 Appendix — (DOCX) [file pone.0239447.s001.docx]

**Verification of Ansys user subroutine for media material model**

Simulation of uniaxial tension tests of media layer of human anterior descending coronary artery. Experimental data, model parameters and reference analytical prediction adopted from [1]:

Material parameters:

- µ = 2.31 kPa
- k_1_ = 8.45 kPa
- k_2_ = 12.84 kPa
- φ = 24.9°
- ρ = 0.3
- (d = 0.00001 kPa^-1^)

Loading:

- Stretch λ = 1.0 to 1.4

**Results:**

**S1 Fig:** **Simulation of uniaxial tension test of material model which was implemented into ANSYS via subroutine**. Our results (red) are drawn against originally published data in [1].

1. Holzapfel GA, Sommer G, Gasser CT, Regitnig P. Determination of layer-specific mechanical properties of human coronary arteries with nonatherosclerotic intimal thickening and related constitutive modeling. J Physiol Hear Circ Physiol. 2005;103: 806–808. doi:10.1152/ajpheart.00934.2004.
