# Supplementary figures and images for: Consideration of stiffness of wall layers is decisive for patient-specific analysis of carotid artery with atheroma
Source: PLoS One. 2020 Sep 29;15(9):e0239447. doi: 10.1371/journal.pone.0239447 (PMC7523976; doi:10.1371/journal.pone.0239447)

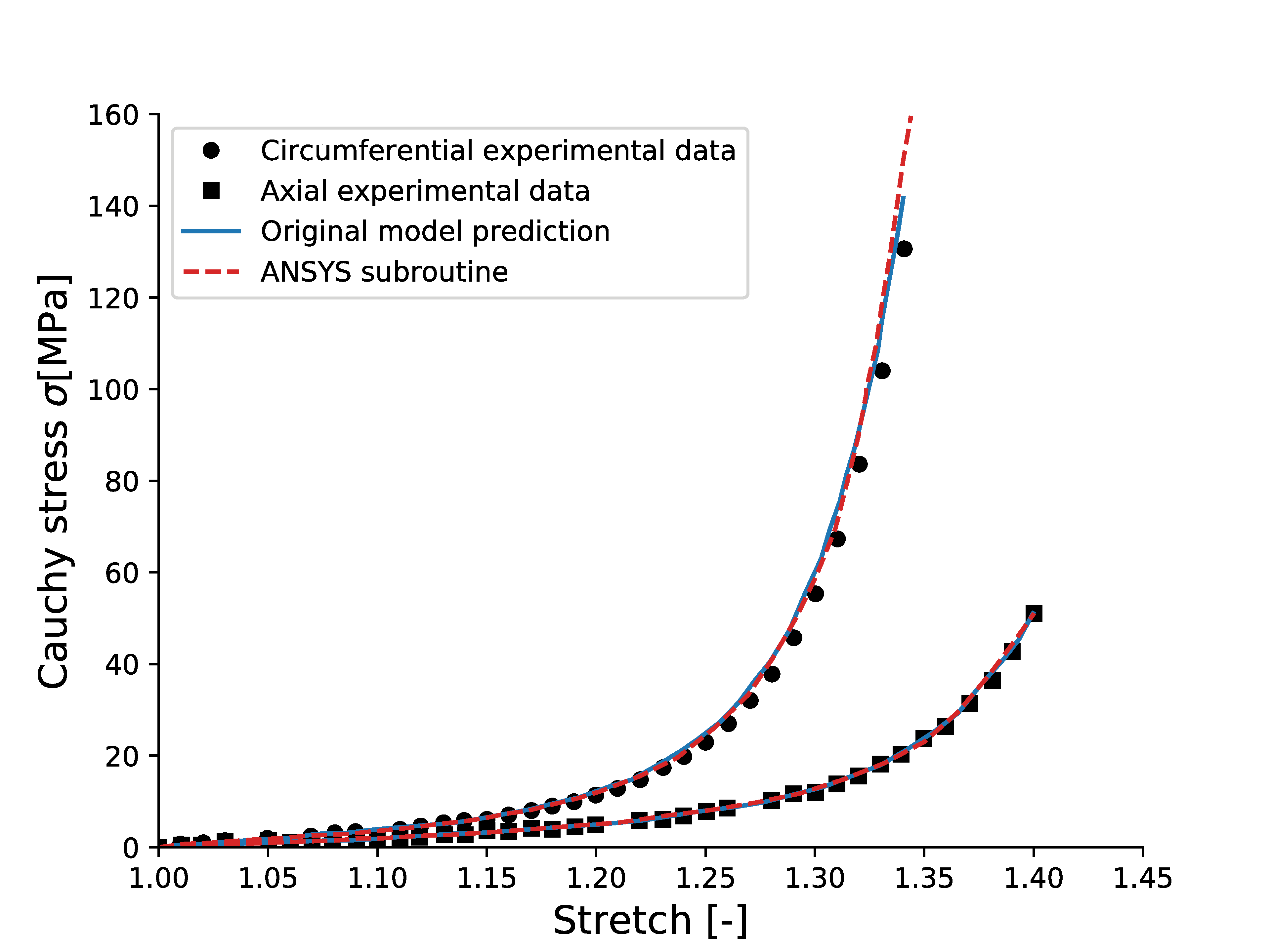

Supplement: S1 Fig — (TIF) [file pone.0239447.s002.tif]

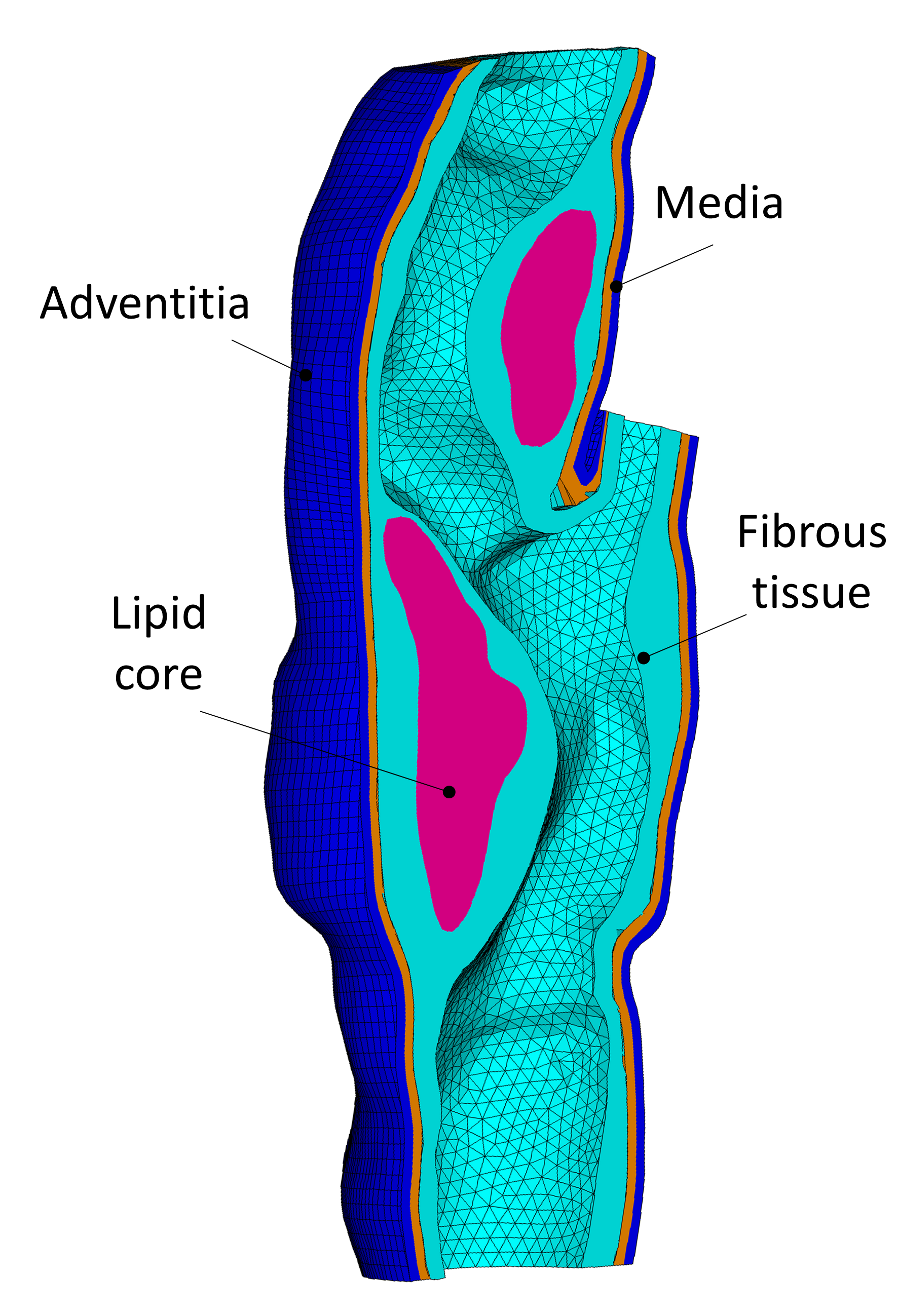

Supplement: S2 Fig — Section of sample 2 model with two large lipid cores. (TIF) [file pone.0239447.s003.tif]
