# Supplementary material for: Consideration of stiffness of wall layers is decisive for patient-specific analysis of carotid artery with atheroma
Source: PLoS One. 2020 Sep 29;15(9):e0239447. doi: 10.1371/journal.pone.0239447 (PMC7523976; doi:10.1371/journal.pone.0239447)
Supplement: S1 File — (PDF) [file pone.0239447.s004.pdf]

Fakultní nemocnice u sv. Anny v Brně
Pracoviště: Etická komise
Pekařská 53, 656 91 Brno, Česká republika
Tel.: +420 543 184195, Fax: +420 543 182999, www.fnusa.cz

FAKULTNÍ NEMOCNICE U SV. ANNY V BRNĚ ®

STANOVISKO ETICKÉ KOMISE K ŽÁDOSTI O SOUHLAS S PROVÁDĚNÍM VÝZKUMNÉHO PROJEKTU
OPINION OF THE ETHICS COMMITTEE ON THE RESEARCH PROJECT

Číslo jednací/*Reference Number:* **12V/2017**

Datum a místo jednání/*Date and Place of EC Session:* FN u sv. Anny v Brně, dne **07.03.2017**

Jméno a pracoviště žadatele/*Name of Investigator and Designation of Research Project Site:*
FN u sv. Anny v Brně, II. chirurgická klinika, Pekařská 53, 656 91 Brno, Česká republika/*Czech Republic*
Ústav mechaniky těles, mechatroniky a biomechaniky (ÚMTMB) FSI VUT v Brně, Technická 2896/2, 616 69 Brno, Česká republika/*Czech Republic*
CEITEC VUT v Brně, Purkyňova 123, 612 00 Brno, Česká republika/*Czech Republic*

Hlavní zkoušející/*PI:* **prof. MUDr. Robert Staffa, Ph.D.**
Spoluzkoušející/*SI:* MUDr. Robert Vlachovský, Ph.D., MUDr. Tomáš Novotný, Ph.D., MUDr. Luboš Kubíček, prof. Ing. Jiří Burša, Ph.D., Ing. Stanislav Polzer, Ph.D., Ing. Kamil Novák, Ing. Vojtěch Man, prof. Ing. Jozef Kaiser, Ph.D., Ing. Tomáš Zikmund, Ph.D.

Datum doručení žádosti o posouzení výzkumného projektu/*Date of Submission of the Application Form:* 22.02.2017

Název výzkumného projektu/*Title of Research Project:*
Posuzování vulnerability (stability) ateromového plátu v arteria carotis pomocí výpočtového modelování

Seznam předložené dokumentace/*List of Submitted Documents:*
Žádost o stanovisko EK FNUSA k plánovanému výzkumnému projektu
Potvrzení z ICRC
Protokol projektu klinického hodnocení, Verze 1.0 Platnost protokolu od 01.03.2017
Informovaný souhlas verze 1.1/2010, platná od 01.03.2017
CV hlavního zkoušejícího: prof. MUDr. Robert Staffa, Ph.D.
CV spoluzkoušejících: MUDr. Robert Vlachovský, Ph.D., MUDr. Tomáš Novotný, Ph.D., MUDr. Luboš Kubíček, prof. Ing. Jiří Burša, Ph.D., Ing. Stanislav Polzer, Ph.D., Ing. Kamil Novák, Ing. Vojtěch Man, prof. Ing. Jozef Kaiser, Ph.D., Ing. Tomáš Zikmund, Ph.D.

Etická komise vydává souhlasné stanovisko s prováděním výzkumného projektu v místě FN u sv. Anny v Brně (rozhodnuto hlasováním)./*The Ethics Committee issued a favorable opinion on the research project in the St. Anne´s Faculty hospital (adjudicate by voting).*

Poznámka: Toto stanovisko Etické komise nenahrazuje souhlas Fakultní nemocnice u sv. Anny v Brně s prováděním výzkumného projektu, bez kterého nelze provádění zahájit.
Note: This Ethics Committee opinion does not substitute the St. Anne´s Faculty hospital approval with research project. This research project can not be started without St. Anne´s Faculty hospital approval.

Dokumentaci prostudoval a předložil člen etické komise/*The documentation reviewed and introduced member of the EC:*
Ing. Zdeněk Handl

Z přítomných členů etické komise se hlasování neúčastnil/*EC members who did not vote:* prof. MUDr. Miroslav Souček, CSc.

Řešitel je povinen zaslat etické komisi: 1. Oznámení o zahájení výzkumného projektu. **2.** Všechny dodatky protokolu před jejich provedením s výjimkou dodatků, určených k eliminaci bezprostředních rizik pro subjekty hodnocení a dodatků administrativního charakteru. Tyto však musí být následně ohlášeny etické komisi. **3.** Zjištěné skutečnosti, které zvyšují riziko subjektů hodnocení nebo výrazně ovlivňují průběh výzkumného projektu, všechny závažné neočekávané příhody a nové informace, které mohou negativně ovlivnit bezpečnost subjektů hodnocení nebo průběh klinického hodnocení. **4.** Od data zahájení výzkumného projektu nejméně 1x ročně zprávu o jeho průběhu. **5.** Zprávu o ukončení výzkumného projektu.
Investigator is obliged to submit to the EC: *1. Information about research project commencement. 2. Any information with significant impact on the conduct of clinical trial and/or resulting in an increased risk for trial subjects; all serious unexpected adverse reactions and new facts that occurred in relation to the conduct of project and that may increase the safety of trial subjects. 4. Annual report every 12 months during conduct of the research project. 5. Finally report of the research project termination.*

V Brně dne/*In Brno, date* 07.03.2017

FAKULTNÍ NEMOCNICE U SV. ANNY V BRNĚ 656 91 BRNO, Pekařská 53 Multicentrická etická komise 1

prof. MUDr. Vladimír SOŠKA, CSc.
předseda Etické komise FN u sv. Anny v Brně/
Chairperson of the Faculty Hospital St. Anne´s EC

Fakultní nemocnice u sv. Anny v Brně, pracoviště: Etická komise/
Department: Ethics Committee
Pekařská 53, 656 91 Brno, Česká republika
Tel.: +420 543 184195, Fax: +420 543 182 999, www.fnusa.cz

FAKULTNÍ NEMOCNICE U SV. ANNY V BRNĚ ®

Seznam členů etické komise a jejich účast na jednání dne 7. 3. 2017/
List of members of the ethics committee and their attendance at the meeting held on 7 March 2017

Jméno a příjmení *First name and surname*	Muž/ Žena *Male/ Female*	Zaměstnání/odbornost *Occupation/specialism*	Zaměstnanec zřizovatele EK* Ano/*Yes* Ne/*No*	Funkce v EK *Role in EC*	Přítomen *Attendance* Ano/*Yes*Ne/*No*	Účastnil se hlasování *Voted* Ano/*Yes* Ne/*No*
Vladimír Soška	Muž/*Male*	Lékař/*Physician*	☒ ☐	Předseda/ *Chairperson*	☒ ☐	☒ ☐
Eliška Dastychová	Žena/*Female*	Lékař/*Physician*	☒ ☐	Člen/*member*	☒ ☐	☒ ☐
Jana Fogašová	Žena/*Female*	Právník/*Jurist*	☒ ☐	Člen/*member*	☐ ☒	☐ ☐
Zdeněk Handl	Muž/*Male*	Technik/*Engineer*	☒ ☐	Člen/*member*	☒ ☐	☒ ☐
Ota Hlinomaz	Muž/*Male*	Lékař/*Physician*	☒ ☐	Člen/*member*	☐ ☒	☐ ☐
Richard Chaloupka	Muž/*Male*	Lékař/*Physician*	☐ ☒	Člen/*member*	☒ ☐	☒ ☐
Věra Kosová	Žena/*Female*	Lékař/*Physician*	☒ ☐	Člen/*member*	☒ ☐	☒ ☐
Lenka Vítovcová	Žena/*Female*	Důchodkyně/*Pensioner*	☐ ☒	Člen/*member*	☒ ☐	☒ ☐
T. Messerschmidt	Muž/*Male*	Referent/*Officer*	☒ ☐	Člen/*member*	☒ ☐	☒ ☐
Miroslav Souček	Muž/*Male*	Lékař/*Physician*	☒ ☐	Člen/*member*	☒ ☐	☐ ☒
Lubomír Spurný	Muž/*Male*	Farmaceut/*Pharmacist*	☐ ☒*	Člen/*member*	☐ ☒	☐ ☐
Stanislav Synek	Muž/*Male*	Farmaceut/*Pharmacist*	☒ ☐	Člen/*member*	☐ ☒	☐ ☐
Tomáš Talach	Muž/*Male*	Lékař/*Physician*	☒ ☐	Člen/*member*	☒ ☐	☒ ☐
Jan Tomčík	Muž/*Male*	Lékař/*Physician*	☒ ☐	Člen/*member*	☐ ☒	☐ ☐
Lenka Veverková	Žena/*Female*	Lékař/*Physician*	☒ ☐	Člen/*member*	☐ ☒	☐ ☐

(pozn: *Zaměstnanec zřizovatele EK/ *Employee of EC appointing authority)*

Složení Etické komise FN u sv. Anny v Brně odpovídá požadavkům ICH GCP. Člen Etické komise bez pracovně-právního vztahu k FN u sv. Anny v Brně: doc. MUDr. R. Chaloupka, CSc., Lenka Vítovcová, PharmDr. L. Spurný/*The composition of the Ethics Committee of St Anne's University Hospital in Brno meets the requirements of the ICH GCP. The following members of the EC are not employed with the hospital: R. Chaloupka, MD, PhD, Lenka Vítovcová and PharmDr L. Spurný.*

Prohlášení etické komise/*Ethics committee's statement*

Multicentrická etická komise Fakultní nemocnice u sv. Anny v Brně posuzuje všechny projekty biomedicínského výzkumu zahrnujícího lidské účastníky Fakultní nemocnice u sv. Anny v Brně, včetně projektů klinických hodnocení jak z hlediska etického tak i medicínského.

Multicentrická etická komise FN u sv. Anny v Brně pracuje podle svých Standardních pracovních postupů, jednacího řádu a v souladu s předpisy ICH GCP. Při posuzování všech projektů se obecně řídí Helsinskou deklarací světové lékařské asociace (WMA), Mezinárodními etickými směrnicemi pro biomedicínský výzkum zahrnující lidské účastníky (připravené Radou pro mezinárodní organizace lékařských věd - CIOMS ve spolupráci se Světovou zdravotnickou organizací - WHO, vydané v Ženevě 1993).

Při posuzování se Multicentrická etická komise FN u sv. Anny v Brně řídí zejména Úmluvou na ochranu lidských práv a důstojnosti lidské bytosti v souvislosti s aplikací biologie a medicíny: Úmluva o lidských právech a biomedicíně, která je publikována ve sbírce mezinárodních smluv pod číslem 96/2001 Sb. m. s., dále pak zákonem č. 378/2007 Sb. o léčivech, ve znění pozdějších předpisů, vyhláškou č. 226/2008 Sb., o správné klinické praxi a bližších podmínkách klinického hodnocení léčivých přípravků, ve znění pozdějších předpisů, zákonem č. 123/2000 Sb., o zdravotnických prostředcích, ve znění pozdějších předpisů a dále zákonem č. 101/2000 Sb., o ochraně osobních údajů, ve znění pozdějších předpisů.

The Multicentre Ethics Committee of Fakultní nemocnice u sv. Anny v Brně (St Anne University Hospital, Brno) reviews all biomedical research projects involving human subjects conducted at the University Hospital, including clinical trial projects both in terms of ethics and medicine. The Multicentre Ethics Committee of Fakultní nemocnice u sv. Anny v Brně (St Anne University Hospital, Brno) works in compliance with its own rules of procedure and the ICH GCP. Its assessments of projects are generally based on the World Medical Association Declaration of Helsinki (WMA) and the International Ethical Guidelines for Biomedical Research Involving Human Subjects prepared by the Council for International Organizations of Medical Services (CIOMS) in cooperation with the World Health Organization (WHO) and issued in Geneva in 1993. Its reviews are based mainly on the Convention for the Protection of Human Rights and Dignity of the Human Being with regard to the Application of Biology and Medicine: Convention on Human Rights and Biomedicine (Convention for the Protection of Human Rights), published in the Collection of International Treaties under 96/2001 Coll., as well as on the Medicinal Products Act No. 378/2007 Coll., as amended, the Decree No. 226/2008 Coll. providing for the Good Clinical Practice and more detailed conditions for clinical trials with medicinal products, as amended, the Act No. 123/2000 Coll. providing for medical devices, as amended, and the Act No. 101/2000 Coll. providing for personal data protection, as amended.
